# What Influences Coital Frequency Among Chinese Men?: A Cross-Sectional Study

**DOI:** 10.1016/j.esxm.2021.100363

**Published:** 2021-06-02

**Authors:** Yali Xiang, Jingxuan Peng, Jianfu Yang, Yuxin Tang, Dongjie Li

**Affiliations:** 1 Health Management Center, The Third Xiangya Hospital of Central South University, Changsha, China; 2 Health Management Center, Fifth Affiliated Hospital of Sun Yat-sen University, Zhuhai, P.R. China; 3 Department of Urology, The Third Xiangya Hospital of Central South University, Changsha, China; 4 Andrology Center, The Third Xiangya Hospital of Central South University, Changsha, China; 5 Department of Urology, Fifth Affiliated Hospital of Sun Yat-sen University, Zhuhai, P.R. China; 6 Guangdong Provincial Key Laboratory of Biomedical Imaging, The Fifth Affiliated Hospital, Sun Yat-sen University, Zhuhai, Guangdong Province, China; 7 Department of Geriatric Urology, Xiangya International Medical Center, Xiangya Hospital, Central South University, Changsha, P.R. China; 8 National Clinical Research Center for Geriatric Disorders, Changsha, P.R.China; 9 Department of Clinical Pharmacology, Xiangya Hospital, Central South University, Changsha, Hunan, P.R. China; 10 Institute of Clinical Pharmacology, Central South University; Hunan Key Laboratory of Pharmacogenetics, Changsha, Hunan, P.R. China

**Keywords:** Coital Frequency, Sexual Activity, Chinese Male Health, Sexual Health, Population Survey, Cross-sectional Study

## Abstract

**Introduction:**

There are many Western reports on factors influencing coital frequency among men. However, no articles could be found about the factors influencing sexual activity among Chinese men.

**Aim:**

The aim of this study was to identify the factors that influence the coital frequency of Chinese men.

**Main Outcome Measures:**

The main outcome measures included self-reported monthly coital frequency, age, occupation, education level, andrology-related scales and dietary habits.

**Methods:**

Data for 1,407 men aged 18–79 years were collected in the Health Management Center of the Third Xiangya Hospital of Central South University from January 2019 to May 2019. The respondents completed the questionnaires independently or with the help of an interviewer (who read or explained the questionnaires to them) to analyse the factors that influence coital frequency.

**Results:**

In the previous 6 months, the sample had a mean monthly coital frequency (±SD) of 4.34 ± 3.18. Univariate logistic regression results indicated that the number of children (*P* = 0.004), IIEF-5 scores (*P* <0.001), EHSs (*P* <0.001) and frequency of milk consumption (*P* = 0.001) were associated with more frequent sexual activity. These statistical associations did not change after further adjustment for age, occupation, and reproductive history. We observed that the frequency of sexual activity showed an increasing trend with a greater number of children, higher IIEF-5 scores, higher EHSs and greater frequency of milk consumption (test for trend, *P*<0.05). Both univariate and multivariate analysis results indicated that the frequency of sexual activity decreased with increasing age (test for trend, P<0.001).

**Conclusion:**

The coital frequency of Chinese men is associated with erectile function, anthropometric parameters, age, occupation, and dietary habits. **Xiang Y, Peng J, Yang J, et al. What Influences Coital Frequency Among Chinese Men?: A Cross-Sectional Study. Sex Med 2021;9:100363.**

## INTRODUCTION

Sexual health is closely related to overall health, and the former can be regarded as a sign of the status of the latter.^1^^,^^2^ Sexual frequency is one of the most important indicators for evaluating sexual health. Studying the frequency of sex can not only aid in the evaluation of sexual health but also guide clinical work. Previous research has found that male sexual activity is closely related to the occurrence of erectile dysfunction,^3^^,^^4^ prostate cancer,^5^^,^^6^ cardiovascular disease,^7^, ^8^, ^9^ and other diseases. Furthermore, epidemiological studies have shown that sexually active populations have more health benefits, and relationship intimacy and sexual behaviour have a beneficial effect on overall health.^10^, ^11^, ^12^, ^13^

At present, there are many Western reports on factors influencing coital frequency among men. The factors considered include anthropometric parameters,^14^, ^15^, ^16^ education level,^17^, ^18^, ^19^, ^20^, ^21^ marital status,^22^ age,^10^ and social status.^23^ Despite important achievements, questions regarding sexual life are still mostly neglected during routine medical consultations. There is no scientific definition and no consensus on average values of sexual activity, generally referring to the frequency of intercourse. In the literature available to date, only surveys with different methods and noncomparative analyses of sexual activity in different male populations can be found.^24^, ^25^, ^26^, ^27^ However, there is currently no research on sexual frequency in the Chinese male population. In the present study, we explored the influencing factors of the frequency of male sexual intercourse from the perspective of healthy men undergoing a physical examination, and identified factors that influenced it among these populations.

## PATIENTS AND METHODS

### Participants

Men participated in a physical examination at the Health Management Center of the Third Xiangya Hospital of Central South University from January 2019 to May 2019. They gave informed consent and could interrupt or withdraw from the interviews at any moment. The inclusion criteria were as follows: (i) 18-80 years old; (ii) underwent complete health check-ups in the Third Xiangya Hospital of Central South University; (iii) physically active and healthy at the time of the study; and (iv) willing to participate in this study.

According to the above standards, 1407 consecutive heterosexual men participated in this study. The age of the subjects was 18−79 years old, with an average age of 38.7±9.4 years. All protocols were approved by the Institutional review board of the Third Xiangya Hospital of Central South University (No. 2019-S252).

### Measures

The questionnaire included the average monthly coital frequency in the past 6 months, personal history (education, occupation, marital and childbirth history, smoking history, drinking history, eating habits), IIEF-5 questionnaire, EHS questionnaire, and the premature ejaculation diagnostic tool (PEDT). Finally, the relevant physical examination data of the subjects were imported.

The data on the frequency of sexual activity are based on the average frequency of sexual activity per month in the past 6 months, as reported by the subjects. Moreover, the subjects also reported their range of sexual intercourse frequency: none, 1–4 times/month, 5–8 times/month, and ≥9 times/month.

Sexual function was assessed by Andrology-related scales, including the IIEF-5, EHS, and PEDT. Erectile dysfunction was assessed by IIEF-5 scores: severe (1–7), moderate (8–11), mild to moderate (12–16), mild (17–21), and no erectile dysfunction (ED) (22–25). The erection hardness was evaluated using the EHS: penis is larger but not hard (i), penis is hard but not hard enough for penetration (ii), penis is hard enough for penetration but not completely hard (iii), and penis is completely hard and fully rigid (iv). Premature ejaculation (PE) was diagnosed by the PEDT: PE (≥11), suspected PE (9–10), and non-PE (≤8).

### Statistical Analysis

All of the questionnaire results were input into the computer after correction; Microsoft EXCEL 2016 version was used to establish a database. Statistical analyses were performed by utilizing SAS statistical software 9.2 (SAS Institute Inc., Cary, NC). Means ± standard deviations were used to describe the quantitative indicators, and frequencies were used to describe the count data. The Cochran-Mantel-Haenszel (CMH) test was used to analyze the coital frequency distribution at different levels (Tables 1, 3, and 4). The coital frequency was dichotomized using cut-offs of 4 occasions of intercourse per month, and an unconditional logistic regression model was employed to analyze the associations between coital frequency and various related factors, such as age, reproductive history, and occupation. All tests were two-sided, and a probability level of *P*<0.05 was considered significant.

**Table 1:** The effect of anthropometric characteristics on sexual frequency

	Number of occasions of sexual intercourse monthly, mean (SD)	None	1−4 times	5−8 times	≥9 times	*P* value
Age (years)						<0.001
18−29	3.8 ± 4.1	65 (29.28%)	84 (37.84%)	51 (22.97%)	22 (9.90%)	
30-	4.9 ± 3.3	37 (6.03%)	279 (45.44%)	235 (38.27%)	63 (10.26%)	
40-	4.6 ± 2.5	4 (1.14%)	202 (57.39%)	121 (34.38%)	25 (7.10%)	
50-	2.8 ± 2.1	20 (9.13%)	167 (76.26%)	27 (12.33%)	5 (2.28%)	
Occupation						0.010
Self-employed	4.97 ± 3.23	7 (4.79%)	73 (50.00%)	45 (30.82%)	21 (14.38%)	
Worker/farmer	3.91 ± 2.63	7 (6.93%)	53 (52.48%)	33 (32.67%)	8 (7.92%)	
Official	4.53 ± 3.26	13 (5.53%)	128 (54.47%)	75 (31.91%)	19 (8.09%)	
Technical	4.27 ± 2.87	36 (9.21%)	201 (51.41%)	128 (32.74%)	26 (6.65%)	
Manager	4.27 ± 2.31	7 (3.54%)	116 (58.59%)	66 (33.33%)	9 (4.55%)	
Clerk	4.37 ± 4.31	37 (15.04%)	125 (50.81%)	54 (21.95%)	30 (12.20%)	
Others	3.69 ± 2.74	19 (21.11%)	36 (40.00%)	33 (36.67%)	2 (2.22%)	
Education level						0.565
Junior high school or below	3.85 ± 3.00	8 (9.88%)	45 (55.56%)	22 (27.16%)	6 (7.41%)	
Senior high school	4.49 ± 2.96	8 (4.44%)	94 (52.22%)	63 (35.00%)	15 (8.33%)	
						
Junior college	4.47 ± 3.72	31 (9.66%)	170 (52.96%)	88 (27.41%)	32 (9.97%)	
Undergraduate	4.31 ± 3.03	58 (9.37%)				
	323 (52.18%)	191 (30.86%)	47 (7.59%)			
Postgraduate	4.26 ± 3.94	21 (10.19%)				
	100 (48.54%)	70 (33.98%)	15 (7.28%)			
Reproductive history						<0.001
No child	3.74 ± 3.66	93 (30.10%)	107 (34.63%)	80 (25.89%)		
	29 (9.39%)					
With one child	4.37 ± 2.96	21 (3.19%)	392 (59.57%)	196 (29.79%)	49 (7.45%)	
With two or more children	4.7 ± 3.07	12 (2.73%)	233 (52.95%)	158 (35.91%)	37 (8.41%)	
WHR						0.008
Normal	4.3 ± 3.30	73 (10.88%)	340 (50.67%)	200 (29.81%)	58 (8.64%)	
Increase	4.47 ± 3.13	25 (5.33%)	267 (56.92%)	140 (29.85%)	37 (7.89%)	
BMI						0.014
Underweight	4.39 ± 5.78	6 (21.43%)	12 (42.86%)	7 (25.00%)	3 (10.71%)	
Normal	4.19 ± 2.97	77 (10.81%)	365 (51.26%)	216 (30.34%)	54 (7.58%)	
Overweight	4.43 ± 3.31	34 (5.87%)	322 (55.61%)	174 (30.05%)	49 (8.46%)	
Obese	4.85 ± 3.41	9 (10.23%)	33 (37.50%)	37 (42.05%)	9 (10.23%)	

## RESULTS

### Anthropometric Characteristics

The main anthropometric characteristics of the study population are presented in Table 1. The frequency of sex was significantly associated with age (*P* <0.001), occupation (*P* = 0.01), reproductive history (*P* <0.001), waist-to-hip ratio (WHR) (*P* = 0.008), and BMI (*P* = 0.0143). The highest levels of coital activity were reported by men who were 30–39 years of age (4.9±3.3), were self-employed (4.97±3.23), had two or more children (4.7±3.07), had a higher WHR (4.47±3.13), and had a BMI reflecting obesity (4.85±3.41).

### Andrology-Related Scales

The Cronbach's alpha score was calculated as 0.69, showing adequate internal consistency. The test-retest correlation coefficients of each item were ≥0.60, indicating excellent stability over time (*P* < 0.001) (Table 2).

**Table 2:** Test–retest correlation coefficients (R) and P values of IIEF-5, PEDT, and EHS

	IIEF-5					PEDT					
Question	1	2	3	4	5	1	2	3	4	5	EHS
R	0.70	0.69	0.70	0.69	0.70	0.62	0.61	0.60	0.60	0.67	0.68
P	<0.001	<0.001	<0.001	<0.001	<0.001	<0.001	<0.001	<0.001	<0.001	<0.001	<0.001

Table 3 describes the relationship between coital frequency and the andrology-related scales, including the IIEF-5, EHS, and PEDT. The frequency of sex was significantly related to the IIEF-5 (*P* <0.001) and EHS (*P* = 0.0057) questionnaires. Those with normal IIEF-5 scores had a mean sexual activity frequency of 5.14±3.47 times/month, and those with EHSs of 4 had a frequency of 4.55±3.36 times/month. In other words, men with normal erectile function have more frequent intercourse.

**Table 3:** The effect of IIEF-5, EHS and PEDT on sexual frequency

	Number of occasions of sexual intercourse monthly, mean (SD)	None	1−4 times/month	5−8 times/month	≥9 times/month	*P* value
IIEF-5						<0.001
Normal						
	5.14 ± 3.47	29 (4.35%)	311 (46.7%)	247 (37.09%)	79 (11.86%)	
Mild	4.18 ± 2.54	23 (4.28%)	322 (59.85%)	162 (30.11%)	31 (5.76%)	
Mild to moderate	3.06 ± 2.47	11 (9.57%)	79 (68.7%)	21 (18.2%)	4 (3.48%)	
Moderate	2.57 ± 2.57	4 (17.39%)	15 (65.22%)	3 (13.04%)	1 (4.35%)	
Severe	0.22 ± 0.8	59 (90.77%)	5 (7.69%)	1 (1.54%)	0 (0%)	
EHS						0.001
i	3.4 ± 3.1	12 (14.46%)	49 (59.04%)	15 (18.07%)	7 (8.43%)	
ii	3.53 ± 2.71	8 (10.81%)	43 (58.11%)	19 (25.68%)	4 (5.41%)	
iii	4.15 ± 2.62	18 (5.52%)	188 (57.67%)	101 (30.98%)	19 (5.83%)	
iv	4.55 ± 3.36	88 (9.52%)	452 (48.92%)	299 (32.36%)	85 (9.2%)	
PEDT						0.139
Non-PE	4.4 ± 3.23	106 (9.37%)	566 (50.04%)	364 (32.18%)	95 (8.4%)	
Suspected PE	4.02 ± 2.6	11 (6.83%)	94 (58.39%)	45 (27.95%)	11 (6.83%)	
PE	4.12 ± 3.36	9 (7.83%)	72 (62.61%)	25 (21.74%)	9 (7.83%)	

### Lifestyle Factors

In addition, we also studied the relationship between lifestyle factors and coital frequency (Table 4). Coital frequency was significantly associated with the consumption of staple foods (*P* = 0.0056), the frequency of milk consumption (*P* = 0.0233), the frequency of fish or seafood consumption (*P* = 0.0007), and the frequency of fruit consumption (*P* = 0.0158). Sexual activity was more frequent among men who consumed more fibre and rice (4.48±3.29), who drank milk weekly/regularly/daily (4.69±3.49), who ate fish or seafood weekly/regularly/daily (4.53±2.8) and who ate fruit weekly/regularly/daily (4.64±3.27).

**Table 4:** The effect of lifestyle on sexual frequency

	Number of occasions of sexual intercourse monthly, mean (SD)	None	1−4 times	5−8 times	≥9 times	*P* value
Smoking						0.640
No	4.25 ± 3.16	62 (9.47%)	346 (52.82%)	199 (30.38%)	48 (7.33%)	
Regular	4.57 ± 3.16	32 (6.53%)	254 (51.84%)	158 (32.24%)	46 (9.39%)	
Ex-regular	3.71 ± 2.86	5 (8.93%)	34 (60.71%)	14 (25.00%)	3 (5.36%)	
Passive	4.2 ± 3.05	7 (8.54%)	45 (54.88%)	23 (28.05%)	7 (8.54%)	
Amount of smoking						0.214
<10 cigarettes/day	4.54 ± 3.01	11 (8.4%)	64 (48.85%)	43 (32.82%)	13 (9.92%)	
10-20 cigarettes/day	4.43 ± 3.04	17 (6.91%)	133 (54.07%)	71 (28.86%)	25 (10.16%)	
>20 cigarettes/day	4.82 ± 3.57	3 (2.29%)	69 (52.67%)	50 (38.17%)	9 (6.87%)	
Alcohol drinking						0.230
No	4.37 ± 3.30	65 (9.52%)	346 (50.66%)	215 (31.48%)	57 (8.35%)	
Regular	4.34 ± 2.90	38 (6.54%)	321 (55.25%)	176 (30.29%)	46 (7.92%)	
Ex-regular	3.47 ± 4.41	3 (15.79%)	12 (63.16%)	3 (15.79%)	1 (5.26%)	
Staple food						0.006
Mainly rice	4.28 ± 3.05	46 (8.35%)	296 (53.72%)	159 (28.86%)	50 (9.07%)	
Fiber and rice	4.48 ± 3.29	22 (4.99%)	245 (55.56%)	146 (33.11%)	28(6.35%)	
Mainly fiber	4.09 ± 2.97	17 (12.5%)	70 (51.47%)	41 (30.15%)	8 (5.88%)	
Hard to tell	3.39 ± 3.18	21 (13.64%)	68 (44.16%)	48 (31.17%)	17(11.04%)	
Drinking milk						0.023
Never/rarely	4.15 ± 3.24	43 (9.19%)	261 (55.77%)	131 (27.99%)	33 (7.05%)	
Monthly	4.4±2.99	48 (7.15%)	355 (52.91%)	214 (31.89%)	54 (8.05%)	
Weekly/regularly/daily	4.69±3.49	15 (10.42%)	63 (43.75%)	49 (34.03%)	17 (11.81%)	
Eating eggs						0.546
Never/rarely/monthly	4.33±3.27	56 (7.92)	384 (54.31)	207 (29.28)	60 (8.49)	
Weekly/regularly/daily	4.37±2.97	50 (8.68)	295 (51.22)	187 (32.47)	44 (7.64)	
Bean product intake						0.225
Never/rarely/monthly	4.44 ± 3.23	71 (7.88)	464 (51.50)	291 (32.3)	75 (8.32)	
Weekly/regularly/daily	4.12 ± 2.91	35 (9.16)	215 (56.28)	103 (26.96)	29 (7.59)	
Fatty meat intake						0.763
Never/rarely/monthly	4.35 ± 3.16	94 (8.27)	602 (52.95)	352 (30.96)	89 (7.83)	
Weekly/regularly/daily	4.33 ± 3.00	12 (8.22)	77 (52.74)	42 (28.77)	15 (10.27)	
Lean meat intake (per day)						0.145
<50 g	4 ± 3.46	21 (10.82)	107 (55.15)	51 (26.29)	15 (7.73)	
50-100 g	4.44 ± 3.04	55 (6.79)	431 (53.21)	262 (32.35)	62 (7.65)	
>100 g	4.31 ± 3.2	30 (10.75)	141 (50.54)	81 (29.03)	27 (9.68)	
Fish or seafood intake						0.001
Never/rarely/monthly	4.3 ± 3.24	101 (9.77)				
	532 (51.45)					
	316 (30.56)					
	85 (8.22)					
Weekly/regularly/daily	4.53 ± 2.7	5 (2.01)				
	147 (59.04)	78 (31.33)	19 (7.63)			
Personal taste						0.533
Bland	4.16 ± 2.87	39 (8.55)				
	255 (55.92)	129 (28.29)	33 (7.24)			
Salty	4.48 ± 3.1	31 (6.98)				
	230 (51.80)	146 (32.88)	37 (8.33)			
Hard to tell	4.42 ± 3.48	36 (9.40)				
	194 (50.65)	119 (31.07)	34 (8.88)			
Fruit intake						0.016
Never/rarely/monthly	4.16 ± 3.05	73 (9.15)	433 (54.26)	234 (29.32)	58 (7.27)	
Weekly/regularly/daily	4.64 ± 3.27	33 (6.80)	246 (50.72)	160 (32.99)	46 (9.48)	
Vegetable intake (per day)						0.060
<100 g	4.34 ± 3.09	34 (10.46)	163 (50.15)	94 (28.92)	34 (10.46)	
100-200 g	4.38 ± 3.04	54 (6.85)	422 (53.55)	257 (32.61)	55 (6.98)	
>200 g	4.25 ± 3.76	18 (10.59)	94 (55.29)	43 (25.29)	15 (8.82)	

### Logistic Regression Analysis

The mean (±SD) coital frequency per month was 4.34±3.18. Table 5 shows the distribution of coital frequency with regard to the different influencing factors. Univariate logistic regression showed that the number of children (*P* = 0.004), the IIEF-5 scores (*P* <0.001), the EHSs (*P* <0.001) and the frequency of milk consumption (*P* = 0.001) were related to an increased frequency of sex. This was confirmed by multivariate analysis after further adjustment for age, occupation, and reproductive history. The number of children was associated with an increased risk of higher sexual frequency, with OR_NO=1_=2.944 (95% CI: 2.113, 4.101) and OR_NO≥2_=3.307 (95% CI: 2.319, 4.761) compared with subjects without children. We observed the same effect of other factors, such as the IIEF-5 score, EHS and frequency of milk consumption.

**Table 5:** Associations between related influencing factors and coital frequency by univariate and multivariate logistic regression analysis

Characteristic	Patients below cutoff	Patients at or above cutoff	Crude OR	P value	Adjusted OR	*P* value
Age						
18−29	115 (19.69%)	107 (13.0%)	Reference		Reference	
30−39	190 (32.53%)	424 (51.52%)	2.398 (1.752,3.283)	<0.001	1.993 (1.396,2.846)	<0.001
40−49	121 (20.72%)	231 (28.07%)	2.052 (1.456,2.891)	<0.001	1.616 (1.081,2.415)	0.192
50+	158 (27.05%)	61 (7.41%)	0.415(0.279,0.617)	<0.001	0.328 (0.209,0.514)	<0.001
		*P for trend*	<0.001		<0.001	
Career						
Self-employed	52 (8.9%)	94 (11.42%)	Reference		Reference	
Worker/farmer	53 (9.08%)	48 (5.83%)	0.501 (0.299,0.840)	0.009	0.626 (0.367,1.068)	0.086
Official+technical+manager+clerk	437 (74.83%)	633 (76.91%)	0.801 (0.559,1.149)	0.228	0.910 (0.628,1.319)	0.618
Others	42 (7.19%)	48 (5.83%)	0.632 (0.370,1.079)	0.093	0.690 (0.397,1.199)	0.188
Reproductive history						
No child	150 (25.68%)	159 (19.32%)	Reference		Reference	
One child	268 (45.89%)	390 (47.39%)	1.373 (1.046,1.802)	0.022	2.944 (2.113,4.101)	<0.001
Two or more children	166 (28.42%)	274 (33.29%)	1.557 (1.160,2.091)	0.003	3.307 (2.319,4.761)	<0.001
		*P for trend*	0.004		<0.001	
BMI						
Underweight	12 (2.05%)	16 (1.94%)	Reference		Reference	
Normal	306 (52.4%)	406 (49.33%)	0.995 (0.464,2.134)	0.990	1.136 (0.521,2.481)	0.748
Overweight	236 (40.41%)	343 (41.68%)	1.090 (0.506,2.346)	0.826	1.304 (0.592,2.871)	0.511
Obese	30 (5.14%)	58 (7.05%)	1.450 (0.608,3.456)	0.402	1.546 (0.634,3.768)	0.338
		*P for trend*		0.136		0.103
WHR						
Normal	288 (60.63%)	383 (57.85%)	Reference		Reference	
Increase	187 (39.37%)	279 (42.15%)	1.122 (0.882,1.426)	0.348	1.377 (1.062,1.786)	0.016
IIEF-5						
Normal						
	189 (32.36%)	477 (57.96%)	Reference		Reference	
Mild	314 (53.77%)	339 (41.19%)	0.428 (0.341, 0.537)	<0.001	0.486 (0.384, 0.616)	<0.001
Moderate	17 (2.91%)	6 (0.73%)	0.140 (0.054,0.360)	<0.001	0.172 (0.066,0.450)	<0.001
Severe	64 (10.96%)	1 (0.12%)	0.006 (<0.001, 0.045)	<0.001	0.006 (<0.001, 0.045)	<0.001
		*P for trend*	<0.001		<0.001	
EHS						
Iv	351 (60.1%)	573 (69.62%)	Reference		Reference	
Iii	136 (23.29%)	190 (23.09%)	1.342 (0.704,2.560)	0.236	0.911 (0.699,1.188)	0.493
Ii	43 (7.36%)	31 (3.77%)	2.601 (1.575,4.298)	0.001	0.512 (0.311,0.841)	0.008
I	54 (9.25%)	29 (3.52%)	3.040 (1.899,4.865)	<0.001	0.402 (0.246,0.655)	<0.001
		*P for trend*	<0.001		<0.001	
Staple food						
Mainly rice	241 (45.64%)	310 (41.11%)	Reference		Reference	
Fiber and rice	165 (31.25%)	276 (36.6%)	1.300 (1.007,1.680)	0.044	1.352 (1.037,1.762)	0.026
Mainly fiber	60 (11.36%)	76 (10.08%)	0.985 (0.675,1.437)	0.936	0.956 (0.649,1.409)	0.820
Hard to tell	62 (11.74%)	92 (12.2%)	1.154 (0.802,1.659)	0.441	1.053 (0.723,1.533)	0.789
Drinking milk						
Never/rarely	224 (42.42%)	244 (32.32%)	Reference		Reference	
Monthly	253 (47.92%)	418 (55.36%)	1.517 (1.194,1.927)	0.001	1.643 (1.282,2.105)	<0.001
Weekly/regularly/daily	51 (9.66%)	93 (64.58%)	1.674 (1.137,2.464)	0.009	1.916 (1.280,2.868)	0.002
		*P for trend*	0.001		0.001	
Fish or seafood intake						
Never/rarely/monthly	434 (82.2%)	600 (79.47%)	Reference		Reference	
Weekly/regularly/daily	94 (17.8%)	155 (20.53%)	1.193 (0.897,1.585)	0.225	1.300 (0.967,1.746)	0.082
Fruit intake						
Never/rarely/monthly	355 (67.23%)	443 (58.68%)	Reference		Reference	
Weekly/regularly/daily	173 (32.77%)	312 (41.32%)	1.445 (1.146,1.823)	0.002	1.499 (1.180,1.905)	0.001

The test for trend analysis indicated that with a higher number of children, a higher IIEF-5 score, a higher EHS, and a greater frequency of milk consumption, sexual frequency showed an increasing trend (test for trend, *P*<0.05). Both univariate and multivariate analysis results demonstrated that age was associated with the frequency of intercourse (*P*<0.001). The test for trend analysis indicated that coital frequency had a decreasing trend with increasing age (*P*<0.001). However, the average monthly sexual frequency in the 18–30 age group was lower than that in the 30–50 age group. Interestingly, although WHR was not significantly associated with sexual frequency in the univariate analysis, we observed a significant association in the multivariate analysis. Men with an increased WHR (4.47±3.13) had a significantly higher coital frequency than those with a normal WHR (4.2±3.3).

## DISCUSSION

Healthy sexual expression is related to male happiness, health, and overall quality of life.^28^^,^^29^ Coital frequency, one component of sexuality, has been shown to be associated with a number of benefits for physical health.^30^, ^31^, ^32^, ^33^, ^34^, ^35^ It has been proven that men with an active sex life are healthier and happier^36^^,^^37^ and have better cognitive ability^38^ and life expectancy.^39^ The apparent protective role of sexual activity for health may be attributable, at least in part, to the release of endorphins during sexual activity. Endorphin levels are associated with higher natural killer cell activity.^40^ The evidence shows that sexual activity might help prevent infection by bolstering immune function^41^^,^^42^ and protect against cardiovascular disease by lowering the heart rate and blood pressure.^43^ A decreased frequency of sex often indicates a deterioration of physical health^44^ and may also be a predictor of depression and marital disharmony.^45^, ^45^, ^45^, ^46^, ^47^, ^48^

The results of this investigation demonstrate that the mean frequency of sexual activity was 3.8±4.1 times/month among men 18−29 years old, 4.9±3.3 times/month among men 30−39 years old, 4.6±2.5 times/month among men 40−49 years old, and 2.8±2.1 times/month among men over 50 years old. Previous studies have proven that sexual activity tends to decline with age.^49^^,^^50^ In this study, both univariate and multivariate analysis results indicated that sexual frequency declined with age (test for trend, *P*<0.001). However, the sex frequency of the 18- to 30-year-old male population was lower than that of the 30−50-year-old male population, which can be attributed to the lack of stable marriage partners. It has been proven that sexual activity increases with marriage.^51^ However, according to data from the Ministry of Civil Affairs, the marriage rate among young Chinese people has dropped sharply in recent years, which has led to this result.

The frequency of sex was higher among men with a BMI indicating obesity (4.85±3.41) and an increased WHR (4.47±3.13). The results of the multivariate analysis found that the frequency of sex increases with WHR. However, Western studies have reported that body mass index showed a trend towards a negative association with sexual frequency for men,^8^^,^^49^ which is different from our results. Previous research has suggested that sexual activity is not clearly related to other anthropometric parameters and depends mainly on the characteristics of the population examined. Therefore, there is a great need to use similar methods to conduct large-scale studies on larger representative samples worldwide.^14^^,^^15^

Self-employed (4.97±3.23) men have more active sex lives than men in other occupations. It is generally noted that working couples have less time to engage in intimate contact, and married couples in which both partners hold full-time jobs engage in sex less frequently than those in which one partner does not work full time outside the home.^52^ Compared with those in other occupations, those who are self-employed have more disposable time. The test for trend analysis showed that sexual frequency increased with the number of children (*P*<0.05). Men with two or more children had a higher frequency of sex (4.7±3.07) than other men. Donnelly found a positive correlation between the number of children and the frequency of sex, which is consistent with our results.^53^

The relationship between lifestyle factors and coital frequency (Table 3) indicates that sexual frequency is significantly related to the structure of the staple food factor (*P* = 0.0056), the frequency of fish or seafood consumption (*P* = 0.0007), and the frequency of fruit consumption (*P* = 0.0158). Sexual activity was more frequent among men who consumed fibre and rice (4.48±3.29), drank milk weekly/regularly/daily (4.69±3.49), ate fish or seafood weekly/regularly/daily (4.53±2.8) and ate fruit weekly/regularly/daily (4.64±3.27). Moreover, the test for trend analysis demonstrated that sexual frequency increased with the frequency of milk consumption (*P*<0.05). It can be seen from these results that these men follow regular diets and pay attention to dietary health and dietary diversity. Previous studies proved that adherence to the Mediterranean diet (including high consumption of legumes, vegetables and fruits and limited consumption of red meat and dairy products) also improved the frequency of sexual intercourse and significantly reduced the prevalence of sexual dysfunction,^54^^,^^55^ which is consistent with our results.

### Limitations

These data come from a cross-sectional study, and there was no assessment of differences in factors such as changes over time in lifestyle habits, weight, and occupation. As mentioned above, mental status and marital status between partners will affect male sexual activity. Unfortunately, this study did not evaluate psychiatric diagnosis/interventions and marital status. Statistical significance of this study is set at 5% there is a 1 in 20 chance that each comparison will be spurious and driven by chance alone. Given the number of comparisons in this manuscript there is almost certainly at least 1 if not more type 1 errors.

## CONCLUSION

At present, there is a lack of studies on factors related to the frequency of intercourse among Chinese men. In the current study, we found that the coital frequency of Chinese men was associated with erectile function, anthropometric parameters, age, occupation and dietary habits. Therefore, large-scale studies worldwide on larger representative samples are necessary, and more data are needed from other cultures and different age groups.

## STATEMENT OF AUTHORSHIP

Yali Xiang: Conception and Design, Acquisition of Data; Dongjie Li: Conception and Design, Drafting the Article, Revising It for Intellectual Content; Jingxuan Peng: Analysis and Interpretation of Data; Yuxin Tang: Analysis and Interpretation of Data; Jianfu Yang: Analysis and Interpretation of Data; Jingxuan Peng: Drafting the Article; Yuxin Tang: Final Approval of the Completed Article.
